# Coordinated nuclease activities counteract Ku at single-ended DNA double-strand breaks

**DOI:** 10.1038/ncomms12889

**Published:** 2016-09-19

**Authors:** Pauline Chanut, Sébastien Britton, Julia Coates, Stephen P. Jackson, Patrick Calsou

**Affiliations:** 1 Institut de Pharmacologie et de Biologie Structurale, Université de Toulouse, CNRS, UPS, 31077 Toulouse, France; 2 Equipe labellisée Ligue Nationale Contre le Cancer; 3 The Wellcome Trust and Cancer Research UK Gurdon Institute, University of Cambridge, Tennis Court Road, Cambridge CB2 1QN, UK; 4 The Wellcome Trust Sanger Institute, Hinxton, Cambridge CB10 1SA, UK

## Abstract

Repair of single-ended DNA double-strand breaks (seDSBs) by homologous recombination (HR) requires the generation of a 3′ single-strand DNA overhang by exonuclease activities in a process called DNA resection. However, it is anticipated that the highly abundant DNA end-binding protein Ku sequesters seDSBs and shields them from exonuclease activities. Despite pioneering works in yeast, it is unclear how mammalian cells counteract Ku at seDSBs to allow HR to proceed. Here we show that in human cells, ATM-dependent phosphorylation of CtIP and the epistatic and coordinated actions of MRE11 and CtIP nuclease activities are required to limit the stable loading of Ku on seDSBs. We also provide evidence for a hitherto unsuspected additional mechanism that contributes to prevent Ku accumulation at seDSBs, acting downstream of MRE11 endonuclease activity and in parallel with MRE11 exonuclease activity. Finally, we show that Ku persistence at seDSBs compromises Rad51 focus assembly but not DNA resection.

## Abstract (web summary)

Homologous recombination requires end resection of the DNA at the site of the break, however the Ku dimer can sequester single-ended double-strand breaks. Here the authors show that ATM-dependent phosphorylation of CtIP, along with the actions of Mre11, impair the stable loading of Ku onto DNA.

## Graphical abstract

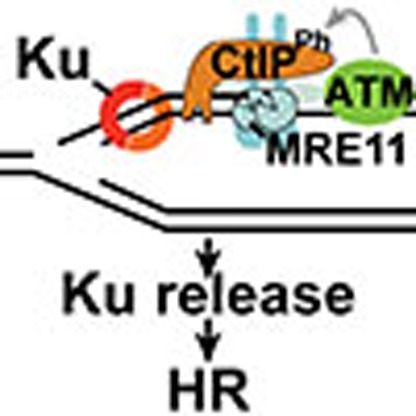

Even a single DNA double-strand break (DSB) is a major threat to genome integrity, since it creates two new uncapped chromosomal DNA ends. In the absence of DNA repair, a DSB can lead to loss of a chromosome or chromosomal fragment and eventually cell death, while if inaccurately repaired, it can promote mutations or large DNA rearrangements such as translocations that can contribute to cell transformation^1^. As part of genome maintenance mechanisms, two main pathways have evolved to repair DSBs in human cells: non-homologous end joining (NHEJ) and homologous recombination (HR)^2^. NHEJ comprises direct ligation of the DSB ends^3^ and is initiated by the DNA termini being recognized by the Ku heterodimer, a highly abundant nuclear protein with strong affinity for a double-stranded DNA end that is threaded into its cavity^4^. Ku bound at a DNA end then recruits the protein PAXX (refs 5, 6) plus the catalytic subunit of the DNA-dependent protein kinase (DNA-PKcs)^7^. The complex of Ku and DNA-PKcs at the DNA end forms the DNA-PK, a serine/threonine kinase able to phosphorylate various substrates and which regulates the DNA-end processing by several enzymes before final DSB ligation by the DNA Ligase IV-XRCC4-Cernunnos/XLF complex.

Although several factors such as nuclear architecture, chromatin and transcriptional context influence repair-pathway choice, NHEJ has the capacity to repair any DSB, providing that it comprises two DNA ends^8^,^9^,^10^. By contrast, end joining is not available as a mechanism of repair at single-ended DSBs (seDSBs), owing to the lack of another DNA end to be ligated to and therefore, seDSBs are preferentially repaired by HR. seDSBs can be generated during replication by the collision of progressing replication forks with various DNA lesions^11^. Repair by HR requires a homology template, most often the sister chromatid, thus helping to explain, why HR is restricted to S and G2 phases of the cell cycle. HR is initiated by DNA resection: the exonucleolytic processing of the 5′-end of the break to generate a free 3′-single-stranded overhang that is stabilized by it being coated with replication protein A, a heterotrimeric complex of RPA70, RPA32 and RPA14 (ref. 2). Notably, free double-stranded DNA ends in human cells are known to be rapidly bound by Ku, owing to its high abundance and affinity for DNA termini. In addition to promoting NHEJ, Ku also shields DNA ends from exonucleases^12^,^13^,^14^,^15^.

In the unicellular organisms *Saccharomyces cerevisiae* and *Schizosaccharomyces pombe*, the proteins scSae2 and spCtp1 (homologues of the human CtIP protein) together with the MRE11–RAD50–Xrs2/NBS1 complex initiate DNA resection at DSB ends through MRE11 endonuclease activity creating an adjacent DNA nick that is then processed in the 5′–3′ and 3′–5′ directions by EXO1-DNA2 and MRE11 exonuclease activities, respectively^16^,^17^. The discovery of MRE11 endo- and exonuclease inhibitors has recently supported the existence of a similar mechanism in higher organisms^18^,^19^. This mode of ‘bi-directional resection’ thus helps explain how resection can be initiated in the presence of Ku, but still leaves the issue of how Ku is finally removed from DNA ends to allow HR to proceed. A role for MRE11 endonuclease activity in the process of Ku release has been demonstrated in yeast but remains to be tested in mammalian cells^12^,^20^,^21^,^22^. In addition, while parallels between this mechanism and Spo11 removal from meiotic DSBs have led to the proposal that eviction of Ku from the DNA end is performed by MRE11 exonuclease activity ‘pushing’ it away^16^, this idea remains to be experimentally tested both in yeast and in human cells.

Here, by using our recently published method to monitor Ku accumulation at DSBs^23^, we show that Ku indeed recognizes seDSBs in human cells. We also establish that ATM-dependent phosphorylation of CtIP plus MRE11 endonuclease activity counteract Ku accumulation at seDSBs. Furthermore, we show that downstream of MRE11 endonuclease activity, the epistatic action of MRE11 exonuclease activity and the recently discovered CtIP flap endonuclease activity^24^,^25^ are required to antagonize Ku, and also for efficient RAD51 loading at seDSBs. Our work also provides evidence for a hitherto unsuspected mechanism operating in parallel to MRE11 exonuclease and CtIP flap-endonuclease activities to counteract Ku persisting at seDSBs. We propose that MRE11 endonuclease and exonuclease activities process the DNA flanking Ku, and therefore prime Ku for release by CtIP 5′-flap endonuclease activity. We anticipate that ‘attacking DNA ends from the flanks’ through the coordinated action of CtIP and MRE11 nuclease activities is a general mechanism to repair complex DNA lesions congested with proteins or bulky DNA adducts.

## Results

### Ku transiently binds to seDSBs

**Figure 1:**
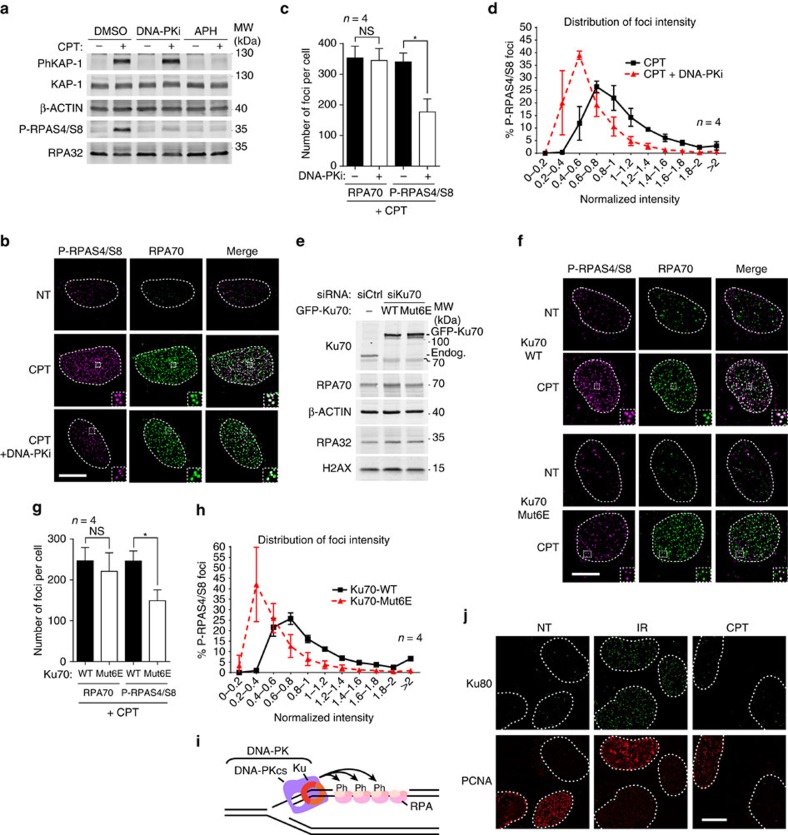
RPA32 S4/S8 phosphorylation is a mark of transient Ku association with seDSBs. (**a**) Immunoblotting of extracts from U2OS cells pre-treated with the replication inhibitor aphidicolin (APH) or with DNA-PK inhibitor (DNA-PKi), respectively, before being CPT treated. (**b**) Representative micrographs of P-RPAS4/S8 and RPA70 foci detected by immunofluorescence in U2OS cells pre-treated with dimethylsulfoxide (DMSO) or DNA-PKi before being treated with CPT. After treatment, cells were pre-extracted with CSK+R (see Methods section) before fixation and immunodetection. (**c**) Quantification of RPA70 and P-RPAS4/S8 foci number per cell. Cells were treated and processed as in **b**, and foci quantified as described in Methods. (**d**) Graph representing the distribution of individual P-RPAS4/S8 focus intensity. Cells were treated and processed as in **b**. 6,684 and 8,462 foci were analysed for the CPT and CPT+DNA-PKi conditions, respectively. (**e**) Immunoblotting of extracts from U2OS T-REx cells stably transfected with control or siRNA-resistant wild-type (WT) or Mut6E GFP-FLAG-Ku70, and transfected with the indicated siRNA. (**f**) Representative micrographs of P-RPAS4/S8 and RPA70 foci detected by immunofluorescence in U2OS T-REx transfected as in **e** and treated with CPT. After treatment, cells were pre-extracted with CSK+R before fixation and immunodetection. (**g**) Quantification of RPA70 and P-RPAS4/S8 foci number per cell. Cells were treated and processed as in **f** and foci quantified as described in Methods. (**h**) Graph representing the distribution of individual P-RPAS4/S8 foci intensity in U2OS T-REx cells treated and processed as in **f**. 9,970 and 19,995 foci were analysed for the Ku70-WT and Ku70-Mut6E conditions, respectively. (**i**) Model depicting the proposed structure transiently forming at seDSB induced by CPT. (**j**) U2OS cells were treated with ionizing radiation (IR) or CPT before being pre-extracted with CSK+R and processed for immunodetection of Ku80 and the replication marker proliferating cell nuclear antigen (PCNA). White scale bars represent 10 μm; insets represent × 3 magnification. Error bars are s.d. Significant differences between specified pairs of conditions, as judged by *t*-test, are highlighted by stars (*P<0.05). NS, non-significant difference.

To decipher the mechanisms regulating Ku binding to and persisting at seDSBs in human cells, we used the topoisomerase I (TopoI) inhibitor and anticancer agent camptothecin (CPT). By stabilizing covalent complexes of TopoI with DNA, CPT promotes the generation of seDSBs associated with replication forks (Supplementary Fig. 1)^26^,^27^. In agreement with previous findings^28^, we observed by immunoblotting that CPT treatment induced phosphorylation of RPA32 on Ser-4/Ser-8 (P-RPAS4/S8) in human U2OS cells in a manner that was abrogated when cells were co-treated with either the specific DNA-PK inhibitor NU7441 (DNA-PKi)^29^ or with the DNA-replication inhibitor aphidicolin (Fig. 1a, note that phosphorylated KAP-1, an ATM target, is still generated in the presence of DNA-PK inhibitor, indicating that DNA-PK inhibition does not affect DSB induction per se). Because DNA-PK activity requires Ku binding to DSBs, these findings implied that an active DNA-PK complex, composed of the Ku70-Ku80 dimer and DNA-PKcs, assembles on and is activated by seDSBs generated by CPT. By using high-resolution imaging, we examined individual seDSBs marked by resection-dependent RPA70 foci and therefore directed towards HR-repair. This revealed that formation of RPA70 foci was accompanied by phosphorylation of S4/S8 RPA32 (P-RPAS4/S8) after CPT treatment (Fig. 1b), supporting the idea that seDSBs are initially recognized by Ku. In agreement with this, inclusion of DNA-PK inhibitor leads to a global decrease of P-RPAS4/S8 foci number and intensity (Fig. 1b–d). Importantly, DNA-PK inhibition had no significant effect on RPA70 foci formation in the same cells, indicating that single-strand DNA (ssDNA) production is essentially unaffected by DNA-PK inhibition (Fig. 1c).

To establish whether S4/S8 RPA32 phosphorylation is indeed a read-out of Ku binding to DNA ends, we replaced endogenous Ku70 by wild-type Ku70 or by the Mut6E mutant of Ku70, which has been shown to be unable to interact with DNA ends^23^ (Fig. 1e). Notably, compared with the wild-type control, replacing of Ku70 by the Mut6E mutant reduced the number and intensity of P-RPAS4/S8 foci without significantly reducing RPA70 foci formation in the same cells, indicating that DNA resection is largely unaffected by the inability of Ku to bind DNA (Fig. 1f–h). Together, these data established that Ku binds to seDSBs generated by CPT treatment, promoting the assembly of a functional DNA-PK complex that mediates RPA32 phosphorylation on S4/S8 (Fig. 1i). Next, we assessed whether Ku persists long enough on seDSBs to allow detection of Ku foci by high-resolution fluorescence microscopy^23^. As published previously^23^, we observed accumulation of Ku (∼250 Ku80 foci per nucleus; see ref. 23 for quantification) at sites of DNA damage caused by ionizing radiation but not at seDSBs induced by CPT (Fig. 1j). Collectively, these data suggested that Ku and DNA-PKcs load transiently on seDSBs as reported by DNA-PK-dependent phosphorylation of RPA32 but that activities prevent stable Ku binding to these DNA ends repaired by HR.

### CtIP prevents Ku accumulation at seDSBs

**Figure 2:**
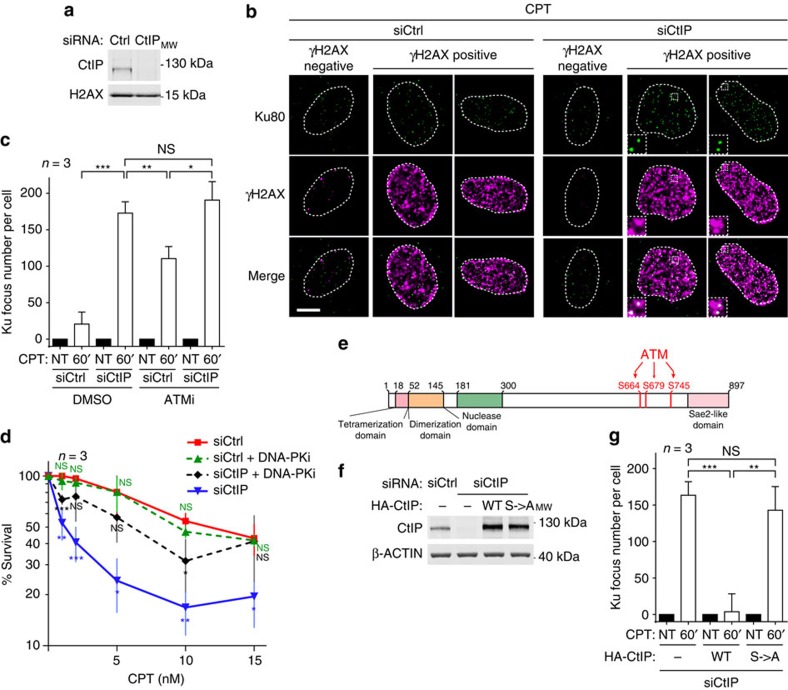
CtIP and ATM-dependent CtIP phosphorylations are required to antagonize Ku at seDSBs. (**a**) Immunoblotting of extracts from U2OS cells transfected with the indicated siRNA. (**b**) Representative micrographs of Ku foci visualized by immunofluorescence in U2OS cells transfected as in **a** and treated with CPT (white scale bar represents 10 μm; insets represent × 3 magnification). (**c**) Quantification of Ku foci in PCNA-positive cells. U2OS cells were transfected with the indicated siRNA and pre-treated with dimethylsulfoxide (DMSO) or ATM inhibitor (ATMi) for 1 h, before being CPT treated and processed for Ku foci detection by immunofluorescence. (**d**) Graph representing cell viability as determined by clonogenical assays of U2OS cells transfected with the indicated siRNA and treated for 18 h with CPT in presence of DMSO or DNA-PK inhibitor. *T*-tests were used to determine significant differences to siCtrl condition. (**e**) Schematic of CtIP domains indicating the position of the mutated ATM-dependent phosphorylation sites. (**f**) Immunoblotting of extracts from U2OS T-REx cells stably transfected with control or siRNA-resistant wild-type (WT) or S→A phosphomutant HA-CtIP expressing plasmids and transfected with the indicated siRNA. (**g**) Quantification of Ku foci in replicating U2OS T-REx cells complemented with HA-CtIP (as in **f**) and CPT treated. Error bars on figures correspond to s.d.’s. Significant differences between specified pairs of conditions, as judged by *t*-test, are highlighted by stars (*P<0.05; **P<0.01; ***P<0.0005). NS, non-significant difference.

Since a role for CtIP homologues scSae2 and spCtp1 in removing Ku from DSBs has been demonstrated in yeast^12^,^20^,^21^,^22^, we next used short-interfering RNA (siRNA) to deplete CtIP from human cells (Fig. 2a). As described previously^29^,^30^, CtIP depletion strongly impaired DNA resection as detected by a flow cytometry-based assay (Supplementary Fig. 2a,b)^30^ without affecting the amount of DSB induced by CPT as revealed by quantitative analysis of the DSB marker phosphorylated histone H2AX (γH2AX, Supplementary Fig. 2c). Strikingly, CtIP depletion also allowed accumulation of Ku foci in replicating cells treated with CPT (see Fig. 2b, right panel, and quantification, Fig. 2c). These findings thus supported there being a critical role for human CtIP in counteracting the accumulation of Ku on seDSBs, as has been reported in yeast for scSae2 and spCtp1 (refs 12, 20, 21, 22). Importantly, we found that the sensitization to cell killing by CPT that is produced by CtIP depletion could be partly rescued by inhibition of DNA-PK (Fig. 2d), suggesting that toxic repair events mediated by NHEJ underlie this sensitization. Under these conditions, DNA-PK inhibition alone had no significant impact on survival of cells transfected with control siRNA, consistent with CPT inducing DNA ends that are normally not repaired by NHEJ but by HR.

### ATM phosphorylates CtIP to prevent Ku persistence at seDSBs

In agreement with our previous findings^23^ and through the use of the specific ATM inhibitor KU55933 (ref. 31), we found that ATM kinase activity also counteracts Ku at seDSBs, although to a lesser extent than CtIP depletion (Fig. 2c). Importantly, since addition of ATM inhibitor did not further increase the number of Ku foci under conditions of CtIP depletion (Fig. 2c), our data strongly suggested that in this regard, ATM and CtIP function in the same pathway. These findings thus suggested that ATM prevents Ku accumulation on seDSBs by stimulating CtIP activity, likely through direct CtIP phosphorylation. To test this, we established a complementation system to express in CtIP-depleted cells, siRNA-resistant wild-type or mutated CtIP, the latter bearing Ser to Ala mutations in three of its main ATM-mediated phosphorylation sites (S664, S679 and S745 (refs 32, 33); Fig. 2e,f). In accord with ATM-mediated CtIP phosphorylation being essential to antagonize Ku accumulation on seDSBs, unlike wild-type CtIP, the phosphorylation site (S->A) mutant CtIP was unable to counteract Ku focus formation in response to CPT (Fig. 2g). These data thereby supported a model in which ATM-mediated CtIP phosphorylation serves to positively regulate the ability of CtIP to prevent Ku from remaining at seDSBs.

### MRE11 nuclease mediates CtIP function in antagonizing Ku

**Figure 3:**
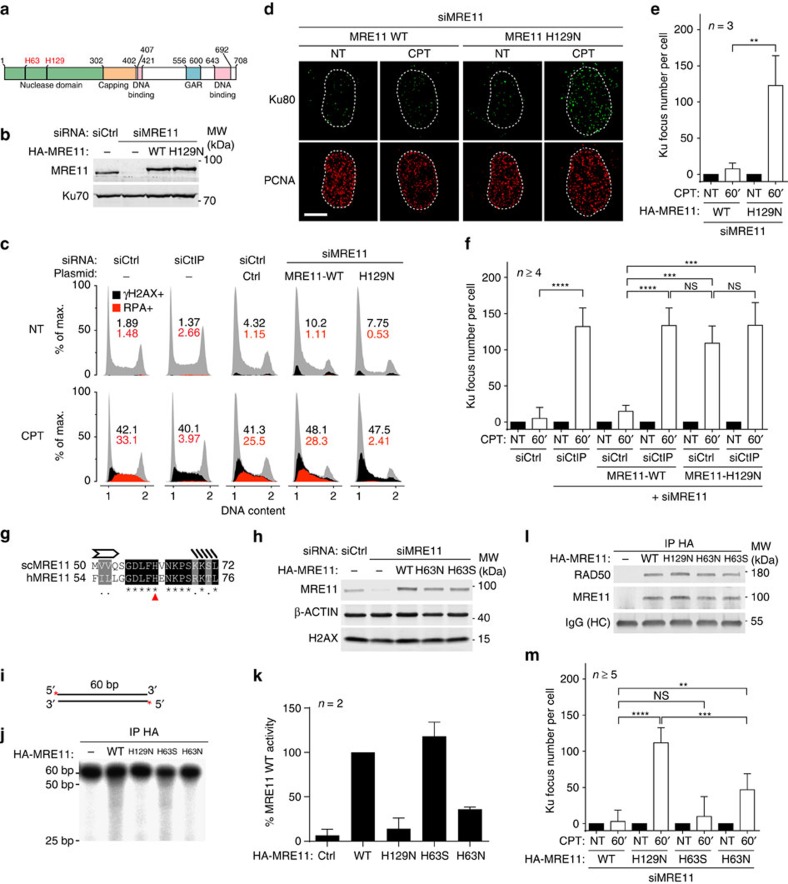
MRE11 nuclease activities control Ku accumulation at seDSB. (**a**) Schematic of MRE11 domains with the position of MRE11 H129 and H63 residues. (**b**) Immunoblotting of extracts from U2OS T-REx cells stably transfected with control or siRNA-resistant wild-type (WT) or H129N HA-MRE11-expressing plasmids and transfected with the indicated siRNA. (**c**) U2OS or U2OS T-REx cells complemented with MRE11 as in **b** were transfected with the indicated siRNA, treated with CPT and processed for analysis of DNA resection as monitored by measuring RPA32 association with chromatin using a flow cytometry assay^30^. (**d**,**e**) Representative micrographs (**d**) and quantification (**e**) of Ku foci in replicating U2OS T-REx cells complemented with MRE11 as in **b** and treated or not with CPT before being processed for immunofluorescence. Replicating cells were identified using proliferating cell nuclear antigen (PCNA) staining. (**f**) Quantification of Ku foci in replicating cells. U2OS T-REx cells stably transfected with control or siRNA-resistant WT or H129N HA-MRE11-expressing plasmids were transfected with the indicated siRNA and Ku foci were quantified in PCNA-positive cells. (**g**) Alignment of scSae2 H59 with human MRE11 H63 revealing that amino acid H59 is conserved in humans and corresponds to H63. (**h**) Immunoblotting of extracts from U2OS T-REx cells stably transfected with control or siRNA-resistant WT, H63S or H63N HA-MRE11-expressing plasmids, and transfected with the indicated siRNA. (**i**) 5′ radio-labelled double-stranded DNA substrate used for *in vitro* nuclease assays. (**j**) Analysis by denaturating PAGE of the exonuclease activity of WT and mutants MRE11 on the probe depicted in **i**. (**k**) Quantification of nuclease activity in each condition relative to the MRE11 WT condition. (**l**) Immunoblotting of bead-associated complexes used for *in vitro* nuclease assays. (**m**) Quantification of Ku foci in replicating U2OS T-REx cells complemented by WT or mutant HA-MRE11 as in **f** and treated with CPT. Error bars are s.d. Significant differences between specified pairs of conditions, as judged by *t*-test, are highlighted by stars (**P<0.01; ***P<0.0005; ****P<0.0001). NS, non-significant difference.

CtIP has been proposed to function together with the MRE11–RAD50–NBS1 complex to promote MRE11 nuclease activity^34^,^35^. We therefore tested the role of MRE11 nuclease activity in counteracting Ku accumulation on seDSBs via replacing endogenous MRE11 by the H129N mutant, which is inactivated for both endo- and exonuclease activities^36^, or by the wild-type MRE11 protein as a control (Fig. 3a,b). In contrast to the wild-type protein, expression of nuclease-inactive MRE11 led to a defect of DNA resection, similar to that caused by CtIP depletion (Fig. 3c), and also to a strong accumulation of Ku foci after CPT treatment (Fig. 3d,e). Importantly, CPT-induced KAP-1 phosphorylation on S824, which is ATM-dependent (Supplementary Fig. 3a), was essentially the same in wild-type versus H129N MRE11 expressing cells (Supplementary Fig. 3b), indicating that ATM activation is not affected by the H129N mutation. These data thus demonstrated that MRE11 nuclease activity is critical to restrain Ku from accumulating at seDSBs generated by CPT and confirmed that it is not required for ATM activation^36^. Furthermore, we found that MRE11 nuclease activity functions in the same pathway as CtIP, since depleting CtIP in cells expressing MRE11 H129N did not further increase the number of Ku foci detected on CPT treatment (Fig. 3f).

To address the respective contributions of MRE11 exo- and endonuclease activities in counteracting Ku accumulation at seDSBs, and since the scMRE11 H59S mutant is selectively deficient in exonuclease activity^16^, we mutated His63, the corresponding residue in human MRE11 to Ser or Asn, and replaced endogenous MRE11 by these mutants or wild-type MRE11 protein (Fig. 3g,h). Through biochemical assays, we found that MRE11 H63N was devoid of 3′–5′ exonuclease activity (Fig. 3i–k), while still being able to associate with RAD50 (Fig. 3l). By contrast, H63S mutant MRE11 behaved essentially as the wild-type protein in exonuclease and RAD50-binding assays. Significantly, in cells expressing H63N, Ku foci accumulated on CPT treatment, to about half the level observed with the nuclease-inactive H129N mutant (Fig. 3m; by contrast, H63S MRE11 nearly fully complemented the effect on Ku focus appearance caused by depleting endogenous MRE11). These data therefore supported a model in which MRE11 3′–5′ exonuclease activity helps to antagonize Ku at about 40% of seDSBs, and also highlighted the existence of another mechanism operating to counteract Ku and in a manner epistatic with MRE11 endonuclease activity.

### Mre11 and CtIP nuclease activities cooperate to counteract Ku

**Figure 4:**
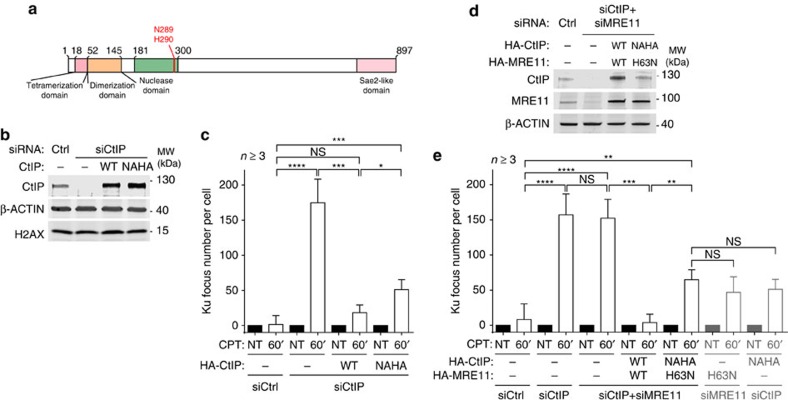
CtIP 5′-flap endonuclease activity shows epistasis with MRE11 3′–5′ exonuclease activity in counteracting Ku binding to seDSBs. (**a**) Schematic of CtIP domains with the position of N289 and H290 residues. (**b**) Immunoblotting of extracts from U2OS T-REx cells stably transfected with control or siRNA-resistant wild-type (WT) or NAHA HA-CtIP-expressing plasmids, and transfected with the indicated siRNA. (**c**) Quantification of Ku focus number in replicating cells transfected as in **b** and treated with CPT. (**d**) Immunoblotting of extracts from U2OS T-REx cells stably transfected with control or siRNA-resistant WT HA-CtIP and HA-MRE11 or NAHA HA-CtIP and H63N HA-MRE11, and transfected with the indicated siRNAs. (**e**) Quantification of Ku foci in U2OS T-REx stably transfected with control or the specified siRNA-resistant constructs and transfected with the indicated siRNAs. Error bars are s.d. Significant differences between specified pairs of conditions, as judged by *t*-test, are highlighted by stars (*P<0.05; **P<0.01; ***P<0.0005; ****P<0.0001). NS, non-significant difference.

It has been recently reported that in addition to its role in stimulating MRE11 nuclease activity, human CtIP also has intrinsic 5′-flap endonuclease activity, similar to that described for scSae2, despite having a different substrate preference^24^,^25^,^37^. To test a potential function of this activity in antagonizing CPT-induced Ku foci, we replaced endogenous CtIP by the N289A H290A (NAHA) mutant, which has been shown to be selectively inactivated for its flap endonuclease activity (Fig. 4a,b)^24^. In contrast to wild-type CtIP, the NAHA mutant exhibited a partial but significant defect in counteracting Ku accumulation at seDSBs (Fig. 4c). To test if this activity was working in parallel with MRE11 exonuclease activity, we replaced endogenous MRE11 and CtIP by wild-type proteins or H63N MRE11 together with NAHA CtIP (Fig. 4d). If MRE11 and CtIP activities act in two parallel pathways, an additive effect on Ku persistence should be observed. However, under these conditions, we observed that the number of Ku foci on CPT treatment was essentially equivalent between cells expressing simultaneously H63N MRE11 and NAHA CtIP proteins (Fig. 4e) and cells expressing these proteins individually (Figs 3g and 4c; also represented in grey in the most right-hand part of Fig. 4e). We therefore concluded that MRE11 exonuclease and CtIP flap endonuclease activities are epistatic and work together to antagonizing Ku accumulation at seDSBs. Our data also indicated that this mechanism operates at ∼40% of seDSBs (Fig. 4e), therefore providing further evidence for an additional mechanism downstream of MRE11 endonuclease activity and in parallel with MRE11 exonuclease and CtIP endonuclease activities.

### Ku at seDSBs impairs RAD51 loading but not DNA resection

**Figure 5:**
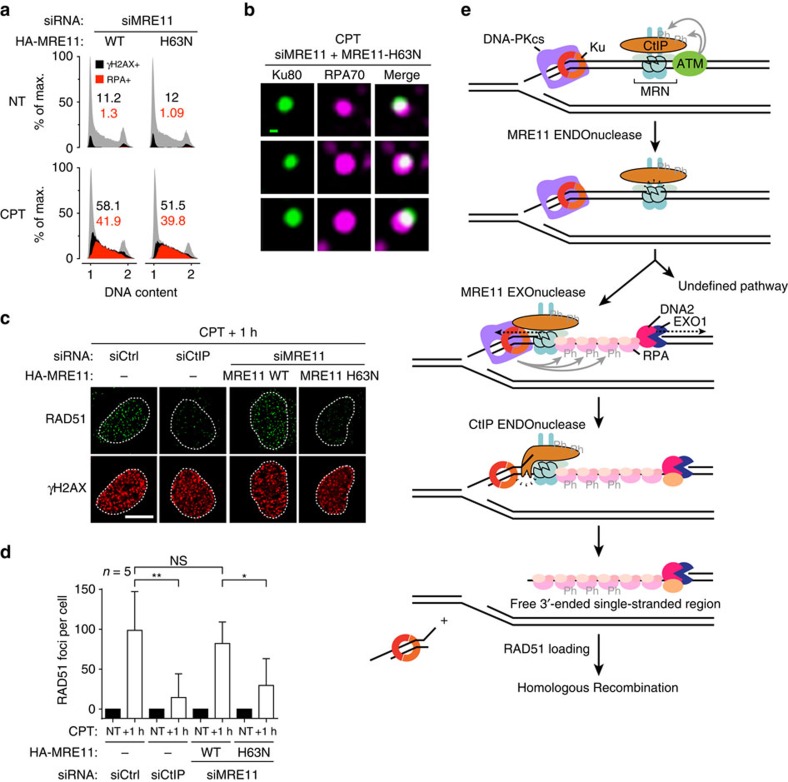
Releasing Ku from seDSBs is critical for RAD51 loading but not for resection. (**a**) Analysis by flow cytometry of DNA damage induction and DNA resection. U2OS T-REx cells stably transfected with siRNA-resistant wild-type (WT) or H63N HA-MRE11-expressing plasmids were transfected with siRNA, treated with CPT and processed for analysis of the γH2AX DNA damage marker and RPA32 association to chromatin. (**b**) Representative micrographs of Ku80 foci showing co-localization with RPA70. U2OS T-REx cells stably transfected with siRNA-resistant H63N HA-MRE11-expressing plasmid were transfected with the indicated siRNA, treated with CPT and processed for immunofluorescence. The green scale bar represents 100 nm. (**c**,**d**) Representative micrographs of RAD51 foci (**c**) and related quantification (**d**) in U2OS T-REx stably transfected with control or siRNA-resistant WT or H63N HA-MRE11-expressing plasmids, transfected with the indicated siRNA, treated with CPT and post-incubated for 1 h in drug-free medium. The white scale bar represents 10 μm. Error bars are s.d. Significant differences between specified pairs of conditions, as judged by *t*-test, are highlighted by stars (*P<0.05; **P<0.01). NS, non-significant difference. (**e**) Proposed model integrating the present findings. Ku and MRN simultaneously recognize seDSBs, with Ku loading at the DNA end and MRN associating to the side of the DSB. Ku recruits DNA-PKcs to form the active DNA-PK complex while MRN recruits ATM that mediates CtIP phosphorylation on multiple residues, including S664, S679 and S745. This activates MRE11 endonuclease activity that creates a nick on the side of the DSB. This nick is extended in the 5′–3′ direction by EXO1 and DNA2 exonuclease activities, which create ssDNA that recruits RPA that is phosphorylated locally on RPA32-S4/S8 by activated DNA-PK. Then, for a subset of seDSBs, MRE11 exonuclease activity processes the DNA flanking Ku and contributes to the generation of a 5′-flap cleaved by CtIP endonuclease activity to release Ku. The generation of a free ssDNA end by this activity is critical for replacement of RPA by RAD51 and HR. Another, as-yet uncharacterized mechanism operates in parallel to release Ku from >50% of seDSBs.

To test the importance of antagonizing Ku at seDSBs for the process of HR, we used MRE11 H63N to induce Ku persistence on a subset of seDSBs. When endogenous MRE11 was replaced by the 3′–5′ exonuclease-inactive H63N mutant DNA resection was essentially unaffected (Fig. 5a), in agreement with the idea that MRE11 exonuclease activity has a minor contribution to resection, probably due to its activity being initiated from a nick close to the DNA end^17^. Importantly, this result also indicated that DNA resection and RPA loading can progress in the presence of Ku. Supporting this idea, we could detect accumulation of Ku80 at 36.5% of RPA70 foci in cells expressing H63N MRE11 (s.d.±2.1, *n*=3; Fig. 5b). However, when RAD51 foci were visualized by immunofluorescence microscopy in the same cells, a clear defect in focus formation was observed in cells expressing H63N MRE11 as compared with cells expressing wild-type MRE11 (Fig. 5c,d). Collectively, these data indicated that DNA resection and RPA recruitment can tolerate Ku persistence at DNA ends, and suggest that effective replacement of RPA by RAD51 requires Ku being cleared away from the DNA end (Fig. 5e).

## Discussion

It has long been clear that after DSB repair, Ku might become trapped on DNA, thereby interfering with DNA transactions such as transcription or nucleotide excision repair^38^,^39^. It was recently found that to circumvent such issues, Ku trapped on DNA after DNA repair is removed in a neddylation-dependent process that promotes its ubiquitylation, chromatin extraction and probably proteasome-mediated degradation^40^,^41^. Ku can also pose another problem when loaded on some DNA ends: while NHEJ-mediated repair is appropriate when two DNA ends are present, it is a dead-end if it occurs on a seDSB generated by the collision of replication forks with a DNA lesion. In such circumstances, Ku could inhibit HR by blocking resection-associated exonuclease activities, and/or promote aberrant ligation of two seDSBs, leading to chromosomal rearrangements^42^.

While the high abundance of Ku and its strong affinity for DNA ends suggested that it could probably load on any DSB, including a seDSB, this idea is still debated. Here by showing that RPA32 phosphorylation on S4/S8 on CPT treatment depends on the ability of Ku to bind DNA and on DNA-PK kinase activity, we establish that Ku is initially loaded on seDSBs. In accord with this, a recent study based on purification of proteins associated with nascent DNA established that Ku associates with replication sites shortly after CPT treatment of human cells^43^. Collectively, these findings have several implications. First, they show that Ku indeed recognizes seDSBs induced by CPT. Second, since RPA loading requires ssDNA formation, and because RPA phosphorylation by DNA-PK necessitates the two factors being close together, resection must actually be initiated in the context of Ku bound to the DNA end. This conclusion is in line with our finding that resection can be essentially normal despite a partial defect in Ku removal caused by an exonuclease-inactive MRE11 mutant (Figs 3m and 5a) and our previous demonstration that Ku and MRE11–RAD50–NBS1 can coexist at the same DNA end^23^. Collectively, the available data support the existence of a ‘bi-directional resection’ mode in human cells that leads to an intermediate structure depicted in the model presented in Fig. 5e in which resection and RPA-bound ssDNA coexist with Ku that is still bound to the adjacent DSB DNA end. Such a model would explain how we found Ku and RPA to co-localize at CPT-induced seDSBs in cells in which MRE11 exonuclease activity was inactivated (Fig. 5b).

Our work provides evidence for the binding of Ku to seDSBs and highlights the importance of deciphering mechanisms regulating its residence at such structures. Here by using a method to monitor Ku DSB loading^23^, we confirmed that Ku is prevented from remaining at seDSBs in human cells by the DNA-nicking endonuclease activity of the CtIP–MRE11 complex (Fig. 3f). Furthermore, through quantitative evaluations, we established that MRE11 exonuclease activity limits Ku persistence at a subset of seDSBs (Fig. 3m). We thus propose a model wherein MRE11 exonuclease activity functions to process the DNA flanking Ku at these seDSBs (Fig. 5e). Since we also obtained evidence that CtIP 5′-flap endonuclease activity is epistatic to MRE11 exonuclease activity (Fig. 4e), we propose that MRE11 exonuclease activity primes the DNA-flanking Ku for cleavage by CtIP flap endonuclease activity, by generating a DNA structure that CtIP has been shown to cleave *in vitro*^24^,^25^ (Fig. 5e). Such a mechanism would thereby promote efficient repair by HR through minimizing the size of the double-stranded DNA region produced by CtIP endonuclease activity and optimizing the extent of ssDNA available for strand invasion^19^. Our work therefore supports strong coordination between MRE11 endo- and exonuclease and CtIP endonuclease activities, a mechanism that is likely to be conserved in other organisms, since scSae2, the CtIP homologue in *S. cerevisiae*, has also been reported to display structure-specific endonuclease activity^24^,^37^. Contrasting with previous models wherein MRE11 exonuclease activity somehow pushes Ku away and off DNA ends^44^, our data support a model in which Ku affinity for DNA ends prevents its simple release through displacement from a subset of DNA ends and instead requires the elimination of a fragment of double-stranded DNA carrying Ku. The generation and fate of such DNA fragments clearly deserve further investigations.

Our work has also highlighted how ATM kinase activity is critical in limiting Ku residence at seDSBs through the phosphorylation of CtIP. How CtIP phosphorylation regulates CtIP activity is still unclear but our data are in agreement with recent work showing that CtIP endonuclease activity depends on ATM-dependent CtIP phosphorylation^24^. The function of ATM in promoting HR-dependent DSB repair was already well documented^45^,^46^,^47^,^48^, despite the fact that in mouse cells, the impact of ATM inhibition on HR repair is not equivalent to loss of ATM protein^49^,^50^. Our work thereby further highlights how targeting ATM kinase could be used to increase the efficiency of CPT derivatives in anticancer treatments through blocking HR-dependent DNA repair processes.

While Ku binding to DNA DSBs represents the first step of NHEJ, our data indicate that it can also be regarded as the first step in HR, with Ku binding protecting DNA ends from unregulated processing and helping to create a situation in which signals are integrated into a ‘decision’ whether the break should be channelled into NHEJ or HR. Supporting this model, previous works in yeast have shown that Ku has a function at seDSBs, and more generally at DSBs, to regulate the timing of DNA-end processing through blocking access of certain factors to DNA ends^13^,^51^,^52^,^53^,^54^. It seems likely that multiple signals promoting HR, such as cyclin-dependent kinase-mediated phosphorylations, the presence or absence of cohesin, chromatin state and nuclear architecture might depend on and/or cooperate with Ku to regulate the balance between NHEJ and HR^8^,^10^,^55^,^56^. While DNA resection can be initiated and performed in the presence of Ku, our findings indicate that the replacement of RPA by RAD51 is impaired by the presence of Ku at the DNA end (Fig. 5c). This suggests that RPA-RAD51 exchange on ssDNA is coupled to the removal of Ku and/or the Ku-associated double-stranded DNA fragment from DNA ends. The mechanism for such coupling clearly deserves exploration through further studies.

Our work also opens new directions for research by providing evidence for an as-yet uncharacterized mechanism working downstream of MRE11 endonuclease activity—and in parallel to MRE11 exonuclease and CtIP flap-endonuclease activities—to mediate Ku release from more than half of seDSBs (Fig. 4e). Several nuclease activities might substitute for MRE11 exonuclease and/or CtIP endonuclease functions. Alternatively, in some cases, Ku might be modified to loosen its affinity for DNA ends, perhaps as mimicked by separation-of-function mutants in yeast^51^ or degraded at seDSB sites by mechanisms related to those shown to mediate Ku release from DNA after DSB repair is complete^40^,^41^. In this regard, we note that the RNF138 E3 ubiquitin ligase has recently been shown to promote HR and to function in the same pathway as MRE11 to promote Ku release^57^,^58^.

Finally, we suggest that coordination of MRE11 and CtIP nuclease activities is likely to operate at various DNA lesions, including ‘masked’ breaks generated by topoisomerase II inhibition, DNA breaks generated during meiosis by Spo11 nuclease and complex DSBs generated by heavy-particle irradiation. In all these cases, DNA end binding by the Ku protein is prevented by bulkiness or complexity at DNA ends, and NHEJ might be unable to proceed. Attacking the DNA ends ‘from the flank’ through MRE11 endonuclease activity may have evolved to deal with these types of DNA lesions, while the ability to cleave the second DNA strand when displacement of the end-blocking lesion is not possible might constitute a second system to free the end for productive repair. This second mechanism might be especially relevant in the repair of complex DNA DSBs that are resected independently of cell cycle phase^59^,^60^,^61^.

## Methods

### Cell culture

U2OS and U2OS T-REx (from American Type Culture Collection and Thermo Fisher Scientific, respectively) were grown in a 5% CO_2_ humidified incubator at 37 °C in Dulbecco’s modified Eagle’s medium supplemented with 10% fetal bovine serum, 2 mM L-glutamine, 100 U ml^−1^ penicillin and 100 μg ml^−1^ streptomycin. Cells were routinely tested for mycoplasma contamination by DNA staining and microscopy, and mycoplasma-free cells were used in all experiments.

### siRNA transfection

A list of siRNAs used in this work is provided in Supplementary Table 1. siRNA transfections were performed with Lipofectamine RNAiMAX (Thermo Fisher Scientific) using the manufacturer’s instructions. Final concentrations of siRNAs were 50 nM in each experiment. Where two siRNAs were used simultaneously, each was used at a concentration of 25 nM. For most experiments, two rounds of transfection were performed at 24 h intervals, and experiments were carried out 48 h after the second transfection. For experiments in GFP-FLAG-Ku70-expressing cells, previously published conditions were used^23^.

### Plasmids

All plasmids generated for complementation experiments are deposited on the Addgene plasmid repository together with fully annotated maps and sequences. Details about plasmid construction are provided in Supplementary Methods together with a table with the oligonucleotides used (Supplementary Table 2).

### Plasmid transfection and stable cell generation

Plasmid transfections were carried out with Lipofectamine 2000 (Thermo Fisher Scientific) according to the manufacturer’s instructions. For stable transfections, 5 μg of DNA and 10 μl of Lipofectamine 2000 were used to transfect 10^6^ U2OS T-REx cells seeded the day before in a 60 mm dish. The day after transfection, various cell dilutions were seeded into 140 mm dishes and puromycin was added at 0.25 μg ml^−1^ for selection. Two to three weeks afterwards, individual clones were isolated and screened.

### DNA damage and drug treatments

DNA-PK inhibitor (NU7441) and ATM inhibitor (KU-55933), both from Tocris Bioscience, were used at 3 and 10 μM, respectively, with a pre-incubation period of 1 h. Aphidicolin (Sigma-Aldrich) was used at 10 μM with a pre-incubation period of 90 min. For inducing protein expression with pICE, doxycycline (Doxy, Clontech) was added at 2 μg ml^−1^ 24 h before treatment, and drug treatments were performed in presence of Doxy. CPT (Sigma-Aldrich) was used at 1 μM for 1 h. Ionizing radiation treatments correspond to a 10 Gy X-ray irradiation, using calibrated irradiators (RX-650; Faxitron) fitted with a 0.5 mm aluminum filter for soft X-rays, followed by a 5 min post-incubation.

### Antibodies

A list of all primary antibodies used in this work together with working conditions is provided in Supplementary Table 3.

### Immunoblotting

For immunoblotting, whole-cell extracts were prepared by scraping cells in SDS-lysis buffer (SLB; 4% SDS, 20% glycerol and 120 mM Tris-HCl (pH 6.8)), boiling 5 min at 95 °C and 10 strokes through a 25 G needle. Lysates were diluted to 5 μg μl^−1^ in SLB. For loading, an equal volume of a solution of 0.01% bromophenol blue and 200 mM dithiothreitol was added to the extracts which were boiled for 5 min at 95 °C. A unit of 40–50 μg of denatured proteins were loaded for each condition and separated on SDS pre-cast gradient 4–12% polyacrylamide TGX gels (Biorad) and transferred on to nitrocellulose membrane (Protran, Whatman). Ponceau S staining was used to confirm homogeneous loading and the membrane was cut into horizontal strips that were subsequently probed with the appropriate primary antibody and appropriate donkey secondary antibodies coupled to IRDye 800CW (LI-COR Biosciences). An infrared imager was used for detection (Odyssey, LI-COR Biosciences). Digital data were processed using Fiji^62^ and cropped using Photoshop CS6 (Adobe). Uncropped scans of the most important blots are provided in Supplementary Fig. 4.

### Immunofluorescence

Cells were seeded on ∼160 μm thick coverslips (VWR International) 24 h before experiments. After treatments, cells were pre-extracted by two incubation of 3 min at room temperature with CSK buffer (10 mM PIPES (pH 7), 100 mM NaCl, 300 mM sucrose, 3 mM MgCl_2_ and 0.7% Triton X-100) containing 0.3 mg ml^−1^ RNase A (CSK+R), except for RAD51 foci for which pre-extractions were performed once with CSK on ice for 5 min. After pre-extraction, cells were washed with PBS and fixed 15–20 min with 2% paraformaldehyde in PBS before being washed three times with PBS. Before staining, cells were permeabilized 5 min with PBS/0.2% Triton X-100, washed with PBS and blocked with PBS/0.1% Tween-20 (PBS-T) containing 5% bovine serum albumin (BSA). Coverslips were incubated 75 min with primary antibodies in PBS-T/5% BSA, then washed with PBS-T and incubated 45 min with appropriate goat secondary antibodies coupled to AlexaFluor 488 or 594 fluorophores (Thermo Fisher Scientific for anti-mouse and rabbit and Abcam for anti-rat) in PBS-T/5% BSA. Note that for RAD51 staining, the primary antibodies were incubated sequentially. After washes in PBS-T and PBS, coverslips were incubated 15–30 min with 1 μg ml^−1^ 4′,6-diamidino-2-phenylindole (DAPI) in PBS. After washes in PBS, coverslips were dipped in water and mounted on glass slides using VectaShield (Vector Labs) mounting medium.

### High-resolution imaging using deconvolution

High-resolution pictures were acquired by imaging z-stacks containing the whole-cell nucleus with a wide-field Deltavision PersonalDV microscope (Applied Precision, 1,024 × 1,024 CoolSNAP HQ or HQ2 camera, z-stack of 0.2 μm interval) equipped with a × 100 UPlanSApo/1.40 oil objective (Olympus). Deconvolutions were then performed with SoftWoRx (Applied Precision) in conservative mode. On all pictures in the manuscript, the white scale bars correspond to 10 μm. For RPA70 and P-RPAS4/S8 staining, micrographs correspond to the projection of the maximum intensity of two adjacent slices, while for other staining they correspond to a single slice.

### Quantification of focus number per cell and focus intensity

For Ku, P-RPAS4/S8, RPA70 and RAD51 foci quantification, cells were pre-extracted and processed for immunofluorescence. Deconvoluted pictures of >10 cells were acquired for each condition and submitted to automated focus detection using the 3D Objects Counter macro of Fiji, with 10 and 300 pixels the minimum and maximum size of foci, respectively, and with a threshold adjusted in each experiment using a positive control^62^,^63^. To identify cells in S-phase, cells were co-stained for proliferating cell nuclear antigen, a protein that accumulates at chromatin in S-phase and persists after CSK+R extraction (Supplementary Fig. 1). For RAD51 and Ku80 foci quantifications, the average number of foci in untreated cells was subtracted from the average number of foci in treated conditions, to concentrate our analysis on the foci induced by CPT. For quantification of P-RPAS4/S8 focus intensity, automated focus detection was used as previously described to measure max intensity of individual foci. To compute the distribution of focus intensity, in each experiment the intensity of individual foci was normalized to the average max intensity, and the frequency distribution was plotted as per cent of the total number of foci. The graphs were then generated out of four independent experiments processed in a similar way.

### Cell survival by clonogenical assays

Cell survival were performed as follows^23^. Briefly, after transfection with siRNA, cells were seeded at low density the day before treatment, pre-incubated with DNA-PK inhibitor or dimethylsulfoxide for 1 h and treated for 18 h with CPT in presence of inhibitor or dimethylsulfoxide before being washed three times. After 10–15 days, cells were stained with crystal violet and the colonies counted. Data were normalized to the untreated conditions to take into account variations in plating efficiency.

### HA-MRE11 immunoprecipitation

After siRNA-mediated depletion of endogenous MRE11, U2OS T-REx cells stably transfected with control plasmid or plasmid expressing wild-type, H129N, H63S or H63N HA-MRE11 were induced for 24 h with Doxy and cell pellets were collected by scraping cells in cold PBS. For each immunoprecipitation (IP), we used 200 μg of proteins from the supernatant obtained after centrifugation 5 min at 21,890 g at 4 °C of cell pellets resuspended and incubated 30 min at 4 °C in IP lysis buffer (20 mM Tris-HCl (pH 7.8), 150 mM NaCl, 1 mM EDTA, 0.5% NP-40 and 0.2 mg ml^−1^ RNase A). The volume of supernatant used was adjusted to 300 μl using IP lysis buffer, and 500 μl IP dilution buffer (20 mM Tris-HCl (pH 7.8), 150 mM NaCl, 1 mM EDTA and 0.05% NP-40) was added to bring NP-40 concentration under 0.2%. Diluted extracts were incubated 4 h at 4 °C with 50 μl of protein G-coupled magnetic beads (Dynabeads M-280; Thermo Fisher Scientific) pre-loaded with 7 μg anti-HA antibody (HA-7; Sigma-Aldrich). The beads were then washed three times with IP wash buffer (20 mM Tris-HCl (pH 7.8), 500 mM NaCl, 1 mM EDTA and 0.05% NP-40) and once with IP dilution buffer. The beads were resuspend in 50 μl dilution buffer, and 5 μl was used for analysing by immunoblotting the amount of HA-MRE11 in each conditions together with the ability of the various MRE11 mutants to interact with RAD50. The rest was used for *in vitro* exonuclease assays. All buffers were supplemented with protease and phosphatase inhibitors (HALT cocktail, Pierce).

### On beads MRE11 exonuclease assay

HA-MRE11 was immunoprecipitated as described above. Beads were then washed once with IP dilution buffer without protease and phosphatase inhibitors and once with exonuclease buffer (25 mM MOPS KCl (pH 7), 10 mM KCl, 5 mM MnCl_2_, 0.05% Tween-20 and 1 mM dithiothreitol). Beads were then resuspended in 50 μl exonuclease buffer containing 1 mM ATP and 0.01 pmol 60-nucleotide-long double-stranded blunt-ended DNA substrate radioactively labelled at both 5′-ends, generated by annealing oligonucleotides ExoProbe-S and ExoProbe-AS previously labelled with γ32P-ATP by the T4 polynucleotide kinase according to the manufacturer’s instructions (Thermo Fisher Scientific). Beads were incubated 3 h at 37 °C with intermittent shaking at 1,400 r.p.m. for 15 s every 2 min (ThermoMixer, Eppendorf). A volume of 50 μl of 2 × proteinase K buffer (100 mM Tris-HCl (pH 7.8), 1% SDS and 2 mM CaCl_2_) was added to the beads, which were then incubated at 95 °C for 4 min. A volume of 1 μl of proteinase K (Euromedex) at 20 mg ml^−1^ was added to the beads that were incubated 30 min at 50 °C with intermittent shaking as described above. The beads were incubated at 95 °C for 4 min and the supernatant was recovered by magnetic separation. A volume of 50 μl of Tris-EDTA was added to the supernatant and DNA was isolated by phenol-chloroform extraction. Ethanol precipitation was then used to isolate DNA from the aqueous phase using 20 μg ml^−1^ yeast tRNA, 0.3 M sodium acetate and 10 mM MgCl_2_ for maximum precipitation efficiency. Dry DNA was then resuspended in Ficoll loading buffer (1 × TBE, 6% Ficoll-400 and 0.02% bromophenol blue), boiled 95 °C for 3 min and separated on a 10% polyacrylamide 7 M urea 1 × TBE gel. The gel was then exposed on a Storage Phosphor Screen (GE Healthcare) that was scanned on a Storm 840 (GE Healthcare). Digital data were processed using Fiji^62^ and cropped using Photoshop CS6 (Adobe). Quantification of the percentage of exonucleolytic products was performed by measuring with Fiji^62^ the integrated signal intensity corresponding to the degradation products over the total integrated intensity in the corresponding lane.

### Flow cytometry analysis of RPA recruitment to chromatin

Analysis of RPA32 association to chromatin was as follows^30^. Briefly, at the end of treatment, cells were collected by trypsination, washed with PBS and pre-extracted by a 10 min incubation on ice in PBS 0.2% Triton X-100 to remove soluble RPA32. Then cells were washed in PBS 1% BSA and fixed for 15 min with 2% paraformaldehyde. After fixation, cells were washed in PBS 1% BSA, incubated 30 min at room temperature in PBS 0.2% Triton X-100, washed in PBS 1% BSA and incubated 1 h at room temperature in PBS-T 5% BSA containing mouse anti-RPA32 and rabbit anti-γH2AX antibodies. Cells were then washed in PBS 1% BSA and incubated 30 min at room temperature in PBS-T 5% BSA containing goat anti-mouse and anti-rabbit secondary antibodies coupled to AlexaFluor 488 and AlexaFluor 647, respectively, diluted at 1/200. Cells were then washed with PBS 1% BSA and incubated in PBS containing 0.25 mg ml^−1^ RNase A and 2 μg ml^−1^ DAPI. A minimum of 30,000 cells were analysed on a BD LSR II flow cytometer (Becton Dickinson). Data were analysed and formatted using FlowJo v8.8.7. Black and red numbers on each flow cytometry profile correspond to the percentage of γH2AX and RPA32 positive cells, respectively.

### Statistical analysis

When statistical analyses were required, an unpaired two-tailed Student’s *t*-test was performed using GraphPad Prism 5.0 (GraphPad Software) between pairs of conditions. Error bars on figures correspond to s.d.’s. On all figures, significant differences between specified pairs of conditions are highlighted by stars (*P<0.05; **P<0.01; ***P<0.0005; ****P<0.0001). NS stands for non-significant difference.

### Data availability

The plasmids generated for this work have been deposited on the Addgene plasmid repository (Plasmids #82030; #82031; #82032; #82033; #82034; #82035 and #82036). The authors declare that the data supporting the findings of this study are available within the article and its Supplementary Information file, and on request.

## Additional information

**How to cite this article:** Chanut, P. *et al*. Coordinated nuclease activities counteract Ku at single-ended DNA double-strand breaks. *Nat. Commun.* 7:12889 doi: 10.1038/ncomms12889 (2016).

## Supplementary Material

Supplementary Information Supplementary Figures 1-4, Supplementary Tables 1-3, Supplementary Methods and Supplementary References
