# Corrigendum: Coordinated nuclease activities counteract Ku at single-ended DNA double-strand breaks

**DOI:** 10.1038/ncomms15917

**Published:** 2017-06-13

**Authors:** Pauline Chanut, Sébastien Britton, Julia Coates, Stephen P. Jackson, Patrick Calsou

Nature Communications 7: Article number: 12889; DOI: 10.1038/ncomms12889 (2016); Published: 09 19 2016; Updated: 06 13 2017

In this Article, the MRE11 exonuclease mutant H63D is consistently referred to incorrectly as H63N. These errors appear in the Results, Methods, Fig. 3, Fig. 4, Fig. 5, Supplementary Fig. 3, Supplementary Table 2 and Supplementary Methods. The authors sincerely apologize for this mistake.

