# Supplementary material for: Coordinated nuclease activities counteract Ku at single-ended DNA double-strand breaks
Source: Nat Commun. 2016 Sep 19;7:12889. doi: 10.1038/ncomms12889 (PMC5031800; doi:10.1038/ncomms12889)
Supplement: Supplementary Information — Supplementary Figures 1-4, Supplementary Tables 1-3, Supplementary Methods and Supplementary References [file ncomms12889-s1.pdf]

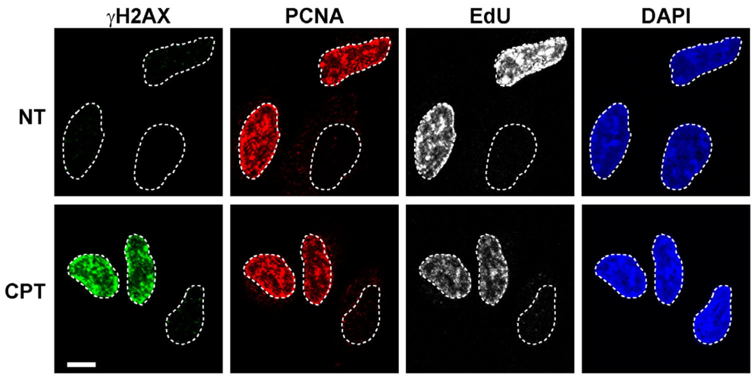

Supplementary Figure 1: CPT induces DSBs specifically in replicating cells that are identifiable by PCNA staining. CPT induces DSBs in replicating cells detected by EdU or PCNA staining after CSK+R pre-extraction. White scale bar = 10 μm.

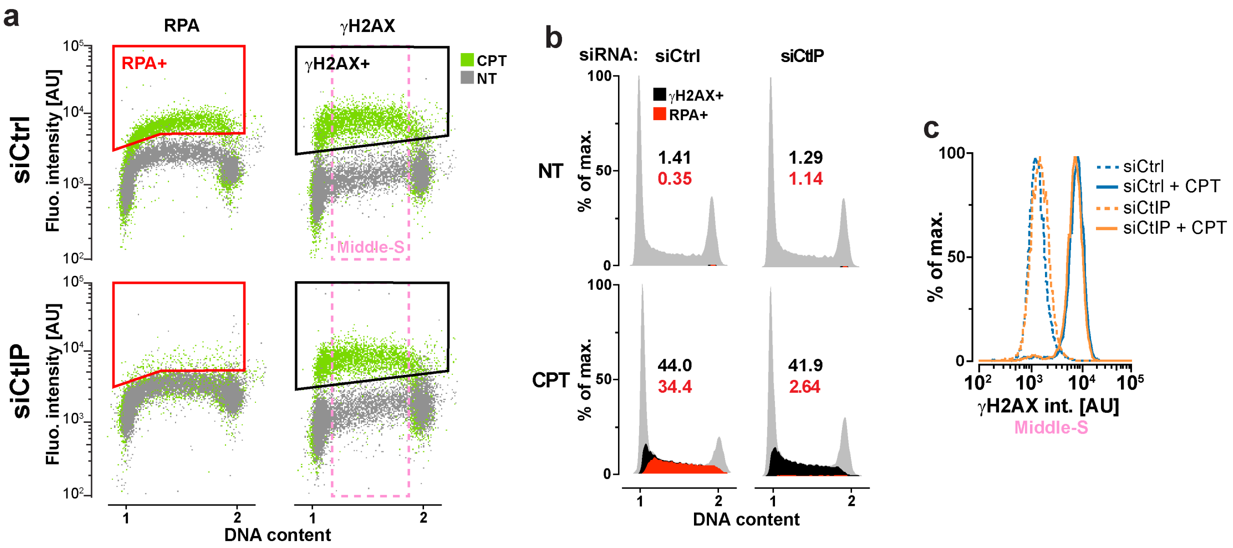

Supplementary Figure 2: CtIP depletion blocks DNA resection without impact on DNA damage production by CPT. (**a**) U2OS cells were transfected with the indicated siRNA, treated with CPT and processed for analysis of γH2AX induction and RPA32 association with chromatin through flow cytometry. The red and black boxes represent the gates used to select RPA positive (RPA+) and γH2AX positive (γH2AX+) cells respectively. The dashed pink box represents the gate used to select cells in middle S phase in which quantification of γH2AX induction presented in **c** were performed. (**b**) Histogram representation of the data presented in **a**. Back and red numbers represent the percentage of γH2AX and RPA32 positive cells, respectively. (**c**) Depletion of CtIP does not impact on the level of DNA damage generated by CPT as revealed by histograms of γH2AX fluorescence intensity.

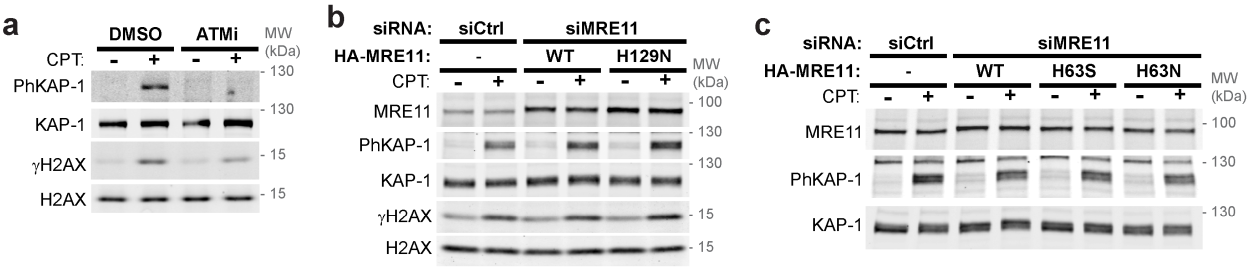

Supplementary Figure 3: ATM-dependent KAP-1 S824 phosphorylation is unaffected by H129N, H63S and H63N MRE11 mutations. (**a**) U2OS cells were treated with CPT in presence of DMSO or ATMi. (**b**) Immunoblotting of extracts from U2OS T-REx stably transfected with control or siRNA-resistant wild-type or H129N HA-MRE11 expressing plasmids, transfected with the indicated siRNA and treated with CPT. (**c**) Immunoblotting of extracts from U2OS T-REx stably transfected with control or siRNA-resistant wild-type, H63S or H63N HA-MRE11 expressing plasmids, transfected with the indicated siRNA and treated with CPT.

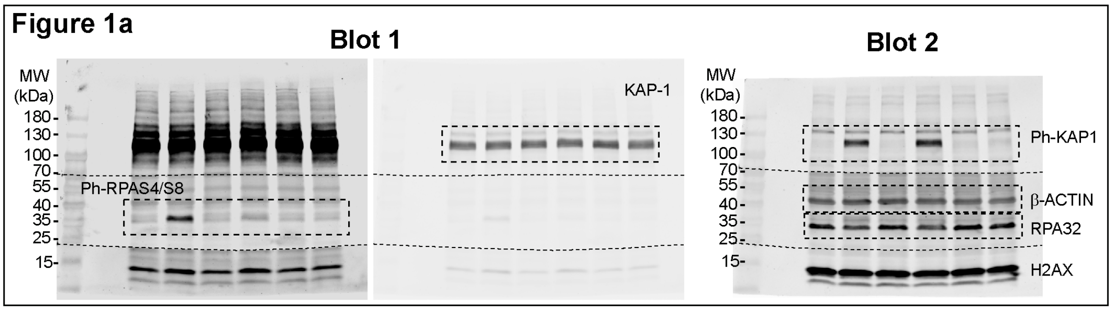

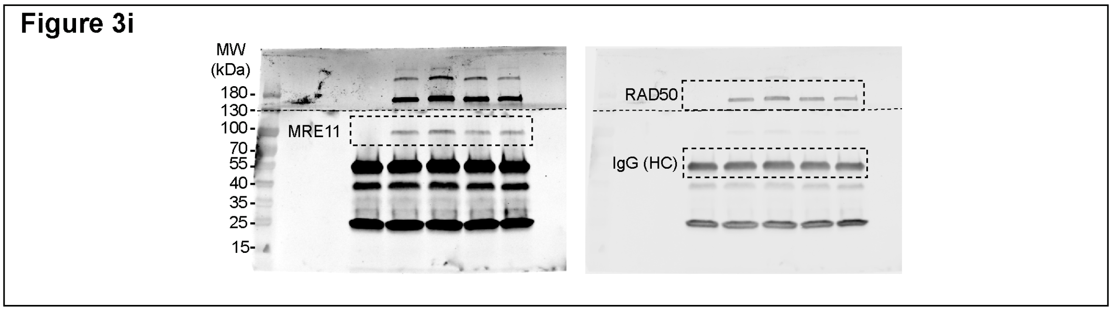

Supplementary Figure 4: Un-cropped scans.

Each panel represents full-length un-cropped blots of the indicated figure. Horizontal dashed lines represent the different stripes of the membranes. When necessary, the same extracts were analyzed onto two different blots as indicated, each including a loading control. The dashed boxes represent the approximate cropped regions displayed in the article.

List of siRNAs used in this study

Target	Target sequence
Control (Firefly luciferase)	CGUACGCGGAAUACUUCGA
CtIP	GCUAAAACAGGAACGAAUC
MRE11 (3'UTR)	GAGCAUAACUCCAUAAGUA
Ku70	GAGUGAAGAUGAGUUGACA

Supplementary Table 1: List of siRNAs used in this study. All siRNAs are terminated by dTdT and are from Sigma-Aldrich.

List of DNA oligonucleotides used in this study

Name	Sequence 5' to 3'	Restriction site(s)
**ExoProbe-S**	*GGGTGAACCTGCAGGTGGGCAAAGATGTCCTAGCAAGGCACTGGTAGAATTCGGCAGCGT	
**ExoProbe-AS**	ACGCTGCCGAATTCTACCAGTGCCTTGCTAGGACATCTTTGCCCACCTGCAGGTTCACCC	
**HA1-S**	AGCTTGCGGCCGCCGCCACCATGTACCCCTACGATGTGCCTGACTACGCCG	HindIII NotI BamHI
**HA1-AS**	GATCCGGCGTAGTCAGGCACATCGTAGGGGTACATGGTGGCGGCGGCCGCA	HindIII NotI BamHI
**CtIP-F**	GCGGGATCCATGAACATCTCGGGAAGCAGCTGTGG	BamHI
**CtIP-R**	GGCACGCGTTTATGTCTTCTGCTCCTTGCCTTTTGG	MluI
**CtIP-S664A-F**	CCGGGAGCAGACCTTGCTCAGTATAAAATGG	
**CtIP-S664A-R**	CCATTTTATACTGAGCAAGGTCTGCTCCCGG	
**CtIP-S679A-F**	GATACAAAGGATGGCGCTCAGTCAAAATTAGG	
**CtIP-S679A-R**	CCTAATTTTGACTGAGCGCCATCCTTTGTATC	
**CtIP-S745A-F**	GGCAGACAGTTTCGCCCAAGCAGCAGATGAAGAG	
**CtIP-S745A-R**	CTCTTCATCTGCTGCTTGGGCGAAACTGTCTGCC	
**CtIP-NAHA-F**	CCACTGTCTGGAAGGAGCCGCCAAGAAACAGCCTTTTGAGGAATC	
**CtIP-NAHA-R**	GGCGGCTCCTTCCAGACAGTGGTAGAGCTCATCACCAAGG	
**HA2-S**	AGCTTGCGGCCGCCGCCACCATGTACCCCTACGATGTGCCTGACTACGCCACCGGTG	HindIII NotI AgeI BamHI
**HA2-AS**	GATCCACCGGTGGCGTAGTCAGGCACATCGTAGGGGTACATGGTGGCGGCGGCCGCA	HindIII NotI AgeI BamHI
**MRE11-F**	GGCACCGGTATGAGTACTGCAGATGCACTTGATG	AgeI
**MRE11-R**	GGCGGTACCTTATCTTCTATTTCTTCTTAAAGAACTAGTGTTCATAAAAGG	KpnI
**MRE11-H129N-F**	GTGTTTAGTATTCATGGCAATAATGACGATCCCACAGGGGCAGATGC	
**MRE11-H129N-R**	GCATCTGCCCCTGTGGGATCGTCATTATTGCCATGAATACTAAACAC	
**MRE11-H63S-F**	GGTGATCTTTTTAGCGAAAATAAGCCCTCAAGGAAAACATTACATACC	
**MRE11-H63S-R**	GGCTTATTTTCGCTAAAAAGATCACCACCTAACAAAATAAAATCC	
**MRE11-H63N-F**	GGTGATCTTTTTGACGAAAATAAGCCCTCAAGGAAAACATTACATACC	
**MRE11-H63N-R**	GGCTTATTTTCGTCAAAAAGATCACCACCTAACAAAATAAAATCC	

Supplementary Table 2: List of DNA oligonucleotides used in this study. * indicates the position of the CF680R fluorophore. All oligonucleotides are from Sigma-Aldrich.

List of primary antibodies used in this study

Target	Clonality	Clone/ Ref.	Raised in	Source	Dilution for I.B.	Dilution for I.F.	Dilution for F.C.
**β-ACTIN**	Monoclonal	ab8226	Mouse	Abcam	1/5000		
**β-ACTIN**	Monoclonal	MAB1501	Mouse	Chemicon	1/200000		
**CtIP**	Monoclonal	14-1	Mouse	Richard Baer	1/50		
**γH2AX**	Polyclonal	2577	Rabbit	Cell signaling	1/500	1/100	1/100
**γH2AX**	Monoclonal	JBW301	Mouse	Millipore	1/1000	1/2000	
**H2AX**	Polyclonal	ab11175	Rabbit	Abcam	1/4000		
**HA-tag**	Monoclonal	HA-7	Mouse	Sigma-Aldrich		Used for IP	
**KAP-1**	Polyclonal	ab10483	Rabbit	Abcam	1/5000		
**PhKAP-1 (S824)**	Polyclonal	IHC-00073	Rabbit	Bethyl laboratories	1/200		
**Ku70**	Monoclonal	N3H10	Mouse	Abcam	1/200		
**Ku80**	Monoclonal	111	Mouse	Thermo Fisher Scientific	1/1000	1/100	
**MRE11**	Monoclonal	18/MRE11	Mouse	BD Biosciences	1/1000		
**PCNA**	Polyclonal	ab18197	Rabbit	Abcam		1/2000	
**RAD50**	Monoclonal	13/B3	Mouse	GeneTex	1/500		
**RAD51**	Polyclonal	H-92	Rabbit	Santa Cruz Biotech.		1/200	
**RPA32**	Monoclonal	9H8	Mouse	Abcam	1/750	1/250	1/200
**RPA32 PhS4/S8 (P-RPAS4/S8)**	Polyclonal	a300-245A	Rabbit	Bethyl laboratories	1/2000	1/2000	
**RPA70**	Monoclonal	EPR3472	Rabbit	Abcam	1/1000		

Supplementary Table 3: List of primary antibodies used in this study. I.B., Immunoblotting; I.F., Immunofluorescence; F.C., Flow cytometry.

SUPPLEMENTARY METHODS

Identification of replicating cells using EdU incorporation

U2OS cells were incubated for 1 h with 10 μM 5-ethynyl-2'-deoxyuridine (EdU) in the presence or absence of CPT. At the end of the treatment, cells were pre-extracted with CSK+R as described and fixed. γH2AX and PCNA detection were performed sequentially and the cells were fixed again after secondary antibody incubation. EdU detection was performed using the EdU click-it kit (Thermo Fisher Scientific) and AlexaFluor647 azide, using manufacturer's instructions. Pictures were acquired on an Olympus FV1000 confocal microscope using a 60x UPlanSApo/1.35 oil objective (Olympus).

DNA manipulations

pICE is a synthetic plasmid conferring puromycin resistance and allowing doxycycline-inducible expression of cDNAs in T-REx cells. The empty pICE control plasmid has been described before (Addgene plasmid #46960)[1]. pICE-HA1 and pICE-HA2 were generated respectively by cloning annealed primers HA1-S/HA1-AS and HA2-S/HA2-AS into pICE digested by HindIII/BamHI. To generate **pICE-HA-CtIP-siR-WT (Addgene #82030)**, siRNA-resistant CtIP sequence was amplified by PCR using pEGFP-C1-CtIP[2] as a template and CtIP-F and CtIP-R as primers. The resulting PCR product was digested by BamHI/MluI and cloned into pICE-HA1 digested by the same enzymes. **pICE-HA-CtIP-siR-S→A (Addgene #82031)** was generated by sequentially mutating CtIP cDNA using pICE-HA-CtIP-siR-WT as original template and primer pairs CtIP-F/CtIP-S664A-R, CtIP-S664A-F/CtIP-R for S664, CtIP-F/CtIP-S679A-R and CtIP-S679A-F/CtIP-R for S679, and CtIP-F/CtIP-S745A-R and CtIP-S745A-F/CtIP-R for S745. The resulting PCR products were fused together using primers CtIP-F/CtIP-R, digested by BamHI/MluI and cloned into pICE-HA1 digested by the

same enzymes. To generate **pICE-HA-CtIP-siR-N289AH290A (Addgene #82032)**, CtIP cDNA was amplified using primer pairs CtIP-F/CtIP-NAHA-R and CtIP-NAHA-F/CtIP-R. The resulting PCR products were fused together using primers CtIP-F/CtIP-R, digested by BamHI/MluI and cloned into pICE-HA1 digested by the same enzymes. To generate **pICE-HA-MRE11-WT (Addgene #82033)**, MRE11 cDNA (IMAGE clone 5181691) was amplified using MRE11-F and MRE11-R primers. The resulting PCR product was digested by AgeI/KpnI and cloned into pICE-HA2 digested by the same enzymes. To generate **pICE-HA-MRE11-H129N (Addgene #82034)**, **pICE-HA-MRE11-H63S (Addgene #82035)** and **pICE-HA-MRE11-H63N (Addgene #82036),** MRE11 cDNA was amplified using primer pairs MRE11-F/MRE11-H129N-R and MRE11-H129N-F/MRE11-R for MRE11-H129N, MRE11-F/MRE11-H63S-R and MRE11-H63S-F/MRE11-R for MRE11-H63S and MRE11-F/MRE11-H63N-R and MRE11-H63N-F/MRE11-R for MRE11-H63N. The pair of resulting PCR products were fused together by PCR using MRE11-F and MRE11-R as primers. The resulting PCR products were digested by AgeI/KpnI and cloned into pICE-HA2 digested by the same enzymes.

SUPPLEMENTARY REFERENCES

1 Britton, S., Coates, J. & Jackson, S. P. A new method for high-resolution imaging of Ku foci to decipher mechanisms of DNA double-strand break repair. *J Cell Biol* **202**, 579-595 (2013).

2 Sartori, A. A. *et al.* Human CtIP promotes DNA end resection. *Nature* **450**, 509-514 (2007).
